# Considerations for pooling real-world data as a comparator cohort to a single arm trial: a simulation study on assessment of heterogeneity

**DOI:** 10.1186/s12874-023-02002-7

**Published:** 2023-08-24

**Authors:** Daniel Backenroth, Trevor Royce, Jose Pinheiro, Meghna Samant, Olivier Humblet

**Affiliations:** 1 Janssen Research & Development, Titusville, USA; 2 Flatiron Health, Inc, 233 Spring Street, New York, NY 10013 USA

**Keywords:** Pooling, Meta-analysis, Real-world data, Oncology, Small population, Heterogeneity, Single-arm trial, Real-world comparator cohort

## Abstract

**Background:**

Novel precision medicine therapeutics target increasingly granular, genomically-defined populations. Rare sub-groups make it challenging to study within a clinical trial or single real-world data (RWD) source; therefore, pooling from disparate sources of RWD may be required for feasibility. Heterogeneity assessment for pooled data is particularly complex when contrasting a pooled real-world comparator cohort (rwCC) with a single-arm clinical trial (SAT), because the individual comparisons are not independent as all compare a rwCC to the same SAT. Our objective was to develop a methodological framework for pooling RWD focused on the rwCC use case, and simulate novel approaches of heterogeneity assessment, especially for small datasets.

**Methods:**

We present a framework with the following steps: pre-specification, assessment of dataset eligibility, and outcome analyses (including assessment of outcome heterogeneity). We then simulated heterogeneity assessments for a binary response outcome in a SAT compared to two rwCCs, using standard methods for meta-analysis, and an Adjusted Cochran’s Q test, and directly comparing the individual participant data (IPD) from the rwCCs.

**Results:**

We found identical power to detect a true difference for the adjusted Cochran’s Q test and the IPD method, with both approaches superior to a standard Cochran’s Q test. When assessing the impact of heterogeneity in the null scenario of no difference between the SAT and rwCCs, a lack of statistical power led to Type 1 error inflation. Similarly, in the alternative scenario of a true difference between SAT and rwCCs, we found substantial Type 2 error, with underpowered heterogeneity testing leading to underestimation of the treatment effect.

**Conclusions:**

We developed a methodological framework for pooling RWD sources in the context of designing a rwCC for a SAT. When testing for heterogeneity during this process, the adjusted Cochran’s Q test matches the statistical power of IPD heterogeneity testing. Limitations of quantitative heterogeneity testing in protecting against Type 1 or Type 2 error indicate these tests are best used descriptively, and after careful selection of datasets based on clinical/data considerations. We hope these findings will facilitate the rigorous pooling of RWD to unlock insights to benefit oncology patients.

**Supplementary Information:**

The online version contains supplementary material available at 10.1186/s12874-023-02002-7.

## Background

In the era of precision medicine, generating insights for rare genomically-defined subpopulations often requires pooling of data from multiple sources [1]. Within oncology, an increasingly common use case is the pooling of real-world data (RWD) from different sources to serve as a real-world comparator cohort (rwCC) for a single-arm clinical trial (SAT) in rare subpopulations for research and to support regulatory decision-making [2–4].

General considerations for combining data from multiple clinical research studies have been described, with pooling individual participant data (IPD) [5, 6] as the preferred method; study-level meta-analysis approaches are also frequently used [7–9]. Testing for heterogeneity in outcomes between studies is a standard step in the pooling process [7, 10], and is critical when creating a rwCC from disparate datasets, as a fundamental assumption of a pooled analyses is that comparison of a SAT to two different rwCC should yield statistically identical results (i.e., no heterogeneity).

However, there remain challenges for establishing best practices for pooling heterogeneous RWD into a rwCC. First, using RWD as a data source for clinical research requires consideration of nuances that may not be as critical with clinical trial data (e.g., differences in quality and organization of the datasets) [11, 12]. Yet, there is no overarching framework, specifically for pooling RWD, that addresses both questions of which studies should be merged (‘what to pool?’), as well as how to integrate them (‘how to pool?’) [13, 14]. Second, standard methods for conducting a heterogeneity assessment are insufficient, as they require unmet assumptions for the rwCC use case; notably, the effect estimates pooled in the meta-analysis are not independent because the SAT is included in all comparisons. Third, the small sample sizes often observed within genomically-defined subpopulations [2] can negatively affect all methods for assessing heterogeneity, with implications for the role of this testing in data analyses, and the interpretation of its results.

To address a gap in published guidance for addressing the above issues, the following provides a framework for the methodologic considerations regarding pooling RWD and specifically for the rwCC use case from the perspective of the sponsor of a novel treatment. Supporting simulations then explore various methods of assessing heterogeneity in outcomes between rwCCs, including an approach that solves issues of non-independence. Conclusions are drawn regarding the strengths and limitations of various heterogeneity tests in different scenarios, and how their results should be interpreted in the context of a rwCC analysis.

### A framework for pooling datasets into a real-world comparator cohort

In the following sections, the steps of the proposed framework describing the pooling of datasets into a rwCC are described, with component steps summarized in Table 1. This framework was developed based on the authors’ review of the literature and experience with data pooling projects.

**Table 1:** Framework for pooling datasets into a real-world comparator cohort

Step	Subcomponent steps
1. Pre-specification	a. A priori research question and processes for statistical analyses and dataset selection
2. Assess dataset eligibility	a. Assess metadata of all datasets & variables for clinical relevance, reliability, and harmonizability
	b. Quantitatively assess non-outcome characteristics and sample size after cohort selection
	c. Determine the eligibility for the final pooled analysis of each dataset according to the prespecified process
3. Outcomes analyses	a. Primary analyses
	b. Assessment of heterogeneity in outcomes
	c. Sensitivity analyses

#### Prespecification of research question and pooling processes

First, the clinical research question should be prespecified [15], followed by preparation of the associated statistical analysis plan. The data requirements for these analyses can then inform the prespecification of the processes for both assessing the eligibility of the candidate datasets for inclusion in the systematic review [16], and whether pooling is appropriate.

#### Assess dataset eligibility

##### Qualitatively assess metadata for all datasets & variables for relevance, reliability, and harmonizability

Before undertaking any assessments related to pooling, each individual dataset that has been identified for detailed assessment by meeting basic eligibility criteria [16] should first be evaluated for its suitability as a potential rwCC given the prespecified requirements. This could include assessment of their fitness-for-use (i.e., relevance and reliability) [17], and suitability as a rwCC to a specific SAT [18, 19]. Interpretation of the assessment results may require complex clinical judgment, however, published guidance is sparse [20].

Once candidate datasets are identified that individually could serve as rwCCs, the feasibility of pooling them must be determined. For this, the prespecified key variables must be harmonizable across datasets [17, 21]. This includes coding them to have comparable values, which typically involves using the lowest common denominator coding scheme, e.g., collapsing pack-years of smoking into ever/never. Such reduction of the granularity of a confounder could lead to residual confounding, in which case investigators may need to weigh the trade-off between bias and increased sample size [21]. In other cases, the values may be comparable but with different definitions and/or ascertainment, in which case pooling could introduce bias. For example, the outcome variable *real-world response*, for which patients without a response assessment are sometimes counted as non-responders and other times excluded.

##### Quantitatively assess non-outcome characteristics and sample size after cohort selection

After applying the relevant inclusion/exclusion (I/E) criteria to each dataset, descriptive analyses can assess the sample size of each dataset, as well as the distributions and missingness of key variables in order to assess their comparability to each other and comparison with a priori expectations (e.g., published literature). If desired and allowable given the pre-specification, the trade-off can be assessed between the strictness of the I/E criteria and the sample size. Prior to conducting this analysis, any participants included in multiple datasets should be identified and deduplicated prior to pooling [22], to avoid double-counting. An a priori hierarchy should determine which data source to use for these participants (e.g., selecting the dataset that optimizes criteria such as data quality and quantity/follow-up time, either for a particular participant or overall).

Finally, after these assessments are complete and the resulting information is synthesized, the eligibility for the final data analysis of each dataset should be determined according to the prespecified process.

#### Outcomes analysis

##### Primary analyses

If it is determined based on the preceding steps that pooling for a particular endpoint is appropriate, then analysis can proceed, treating the real-world datasets as a combined rwCC. If the previous steps provide evidence the individual datasets are not poolable, the sponsor will need to investigate the reasons for any differences [23], as well as analyze and interpret the pooled results appropriately.

##### Heterogeneity assessment

Assessment of heterogeneity is a standard feature of meta-analyses [7]. Extended to the rwCC use case, the fundamental assumption is that comparison of a SAT to two different rwCC should yield statistically identical results (i.e., no heterogeneity). There has not been in-depth consideration of optimal methodologies for testing for heterogeneity in the rwCC use case; this topic is explored further in the remainder of this manuscript. Statistical considerations (described further in the Results) suggest not making the pooling decision solely based on heterogeneity testing, but rather assessing heterogeneity descriptively after the pooling decision has been made based on the considerations for assessing dataset eligibility described above.

##### Sensitivity analyses

A primary benefit of pooling is that it facilitates certain sensitivity analyses that would be infeasible due to sample size limitations if carried out in each dataset separately. These analyses may include assessment of whether intervention effects vary by participant characteristics [24] (e.g., comparing the effect size between different groups, or restricting to a subset of participants after applying additional criteria that increase similarity with the SAT). In principle, heterogeneity should be described separately for each such sensitivity analysis, but if sample size is reduced in each individual dataset after application of these more stringent criteria, then the power of tests of heterogeneity would be decreased.

## Methods

The fundamental assumption of a rwCC analysis is that, after appropriate adjustment for confounders, the SAT-rwCC comparison yields–up to sampling variability–the same estimate that would have resulted from a randomized controlled trial. Given this assumption, comparison of a SAT to two different rwCC should yield identical results up to sampling variability. However, if heterogeneity is detected, this underlying assumption is likely violated, and pooled results should be interpreted with caution. If there are many rwCCs, a meta-analysis using random-effects could be used to summarize the control arms before comparing to the SAT, or to summarize the results of the SAT-rwCC comparisons (although the validity of generating a single summary measure in the presence of heterogeneity is under debate [25]). Here we assume there are only a few rwCCs (e.g., as low as 2 or 3), not enough to make such a meta-analysis feasible [26].

### Methods for testing heterogeneity

We consider two ways in which heterogeneity of outcomes across SAT to rwCC comparisons can be assessed.

First, heterogeneity could be assessed by directly comparing the IPD from the rwCCs (the “IPD” method). Before comparing the rwCCs, it is desirable to make the distribution of prognostic covariates similar between each of these rwCCs and the SAT. This could be accomplished using weighting. Each rwCC would be weighted to the SAT using, for example, average treatment effect on the treated weights. Then the weighted rwCC could be compared to each other using appropriate statistical methods (e.g., using logistic regression if outcomes are binary).

Second, heterogeneity among the effect estimates separately comparing the SAT to each rwCC could be assessed; we call this the aggregate method since it requires aggregate statistics from each SAT-rwCC comparison. This is a traditional non-IPD meta-analysis approach in which the SAT would be compared to each rwCC separately, e.g., using a matching or weighting estimator. Then, the resulting statistics can be assessed for heterogeneity, typically using Cochran’s Q test.

#### A methodology that accounts for dependence: the Adjusted Q-test.

Applying the aggregate method is challenging because the effect estimates being pooled in the meta-analysis will not be independent, since the SAT is included in all comparisons. Therefore, standard methods for assessing heterogeneity, like Cochran’s Q test for heterogeneity, which rely on independence, will be invalid. However, a modified version of Cochran’s Q test can account for this dependence. We call this adjusted test, which is similar to standard methods in the literature for meta-analysis of dependent results [27, 28], the “Adjusted Q-test”. It requires three steps to calculate:

1. In the first step, the covariance matrix Σ of the effect estimates (e.g., risk difference, odds ratio, hazard ratio) from the various SAT-rwCC comparisons is calculated. The effect estimates from the various SAT-rwCC comparisons will be correlated, since each comparison is to the same SAT. One way to calculate this covariance matrix (with dimensions k-by-k, where k is the number of SAT-rwCC comparisons) is to use the bootstrap. A common randomization seed could be used to generate bootstrap samples of the SAT. Then, for each bootstrap sample of the SAT, effect estimates and associated standard errors for each SAT-rwCC comparison could be calculated, using a corresponding bootstrap sample of each rwCC. Then these could be used to calculate the covariance matrix of the effect estimates for the rwCC-SAT comparisons.
2. In the second step, we multiply the effect estimates from the SAT-rwCC comparisons by the inverse Cholesky transformation Σ^−½^. This is a so-called “whitening transformation” which renders the effect estimates uncorrelated, since if the covariance of the effect estimates is Σ, after multiplying those effect estimates by Σ^−½^, their covariance matrix will now be the identity matrix [29].
3. In the third step, Cochran’s Q can be calculated using the resulting statistics, whose assumed covariance matrix is now the identity matrix. Q is then referred to the chi-square distribution with I-1 degrees of freedom, where I is the number of rwCC datasets. This is in accordance with normal practice, although it should be noted that, when, as here, the covariance of the statistics is estimated, using the chi-square distribution is only an approximation to the true distribution of Q and may inflate Type I error. Moreover, the distribution of Q depends on the effect measure i.e., hazard ratios or risk differences [30]. We note that bootstrap methods can be used instead of the chi-square distribution for a potentially more accurate test (see Joshi et al.) [31]. Further work could combine the bootstrap methods of Joshi et al. with the use of the bootstrap above in Step 1.

#### Detecting heterogeneity

We wish to evaluate the impact of the effect sizes on Cochran’s Q and Adjusted Q when assessing heterogeneity of two SAT-rwCC comparisons using a common SAT. We simulate data from two rwCCs and one SAT, using a binary response outcome. We assume the true response rate (RR) is 50% in the SAT and 50% in one of the rwCC (i.e., no difference). However, due to bias from unmeasured confounding, the observed RR in the other rwCC may be different from 50% (i.e., heterogeneity). We calculate the probability that Cochran’s Q test and Adjusted Q will reject the null hypothesis that the effect sizes versus the SAT are the same (i.e., probability of detecting heterogeneity). We compare to the IPD method by also showing the probability that a logistic regression model rejects the null hypothesis that the rwCC arms have the same RR. There are 100 subjects in the SAT and in each rwCC, and we run 1000 replicates for each scenario. For the Adjusted Q test, we generate 1000 bootstrap samples for each replicate.

### Impact of heterogeneity testing

Although potentially inadvisable, one could adopt a conditional approach, where if the above methods did not reveal heterogeneity, then one would proceed with a pooled analysis. This is similar to the test-then-pool approach described by Viele et al. [32]. To explore the operating characteristics of this approach, we consider two hypothetical scenarios of interest. In both scenarios, we assume that a SAT-rwCC comparison is designed using the assumption of an 80% RR in the SAT and 50% RR in the rwCC. To attain 90% power to detect a difference of this size at an alpha level of 5%, roughly 50 participants are required in the SAT and in the rwCC. The sponsor aims to pool two rwCC, each with 25 participants, to achieve the desired power.

#### Scenario 1: impact of heterogeneity testing in null scenario

The first scenario of interest is a null scenario, where the true RR in the SAT and in one of the rwCC is 50%. However, due to some bias (e.g., missing data on patient responses), the RR in the second rwCC may be lower than 50%. To protect against the Type I error inflation that might result from pooling in this scenario, the sponsor pools only if the p-value from an IPD heterogeneity assessment of the two rwCCs is greater than 0.10 (0.10 as opposed to 0.05 is used to increase the power to detect heterogeneity).

#### Scenario 2: impact of heterogeneity testing when treatment improves outcomes

In the second scenario, we consider the RR in the SAT is 80%. The RR in the first rwCC is 50% but due to some bias, the RR in the second rwCC could be higher than 50% (e.g., an unmeasured confounder), potentially leading to Type 2 error.

## Results

### Methods for testing heterogeneity

#### Detecting heterogeneity

**Fig. 1:**
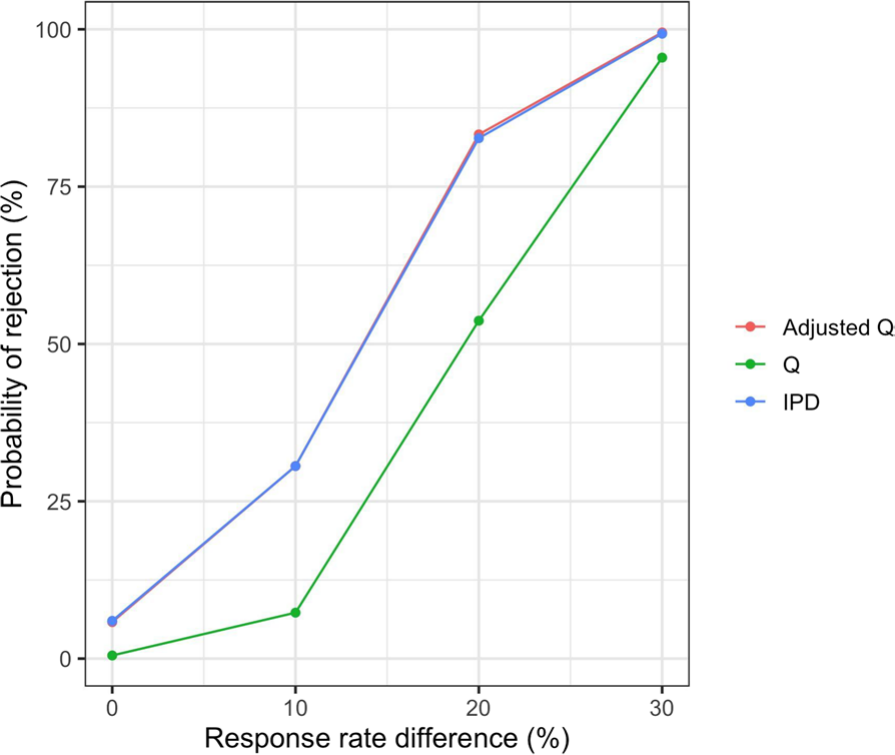
Simulation showing the probability of rejection of the null hypothesis (i.e., of detecting heterogeneity) using Cochran’s Q, Adjusted Q, and the IPD method. For Cochran’s Q and Adjusted Q, two rwCC are separately compared to a SAT and the resulting statistics are assessed for heterogeneity. For the IPD method, the two rwCC are directly compared. 1000 replicates were run for each scenario. For the Adjusted Q test, 1000 bootstrap samples were generated for each replicate. There are 100 subjects in the SAT and in each rwCC

Figure 1 shows the probability that Cochran’s Q test and Adjusted Q will reject the null hypothesis that the effect sizes versus the SAT are the same (i.e., probability of detecting heterogeneity). When rwCCs with identical true RRs are compared to the same SAT, the Type I error of the Q test is lower than the nominal 5% alpha level since there is no variability in the SAT arm across the comparisons. However, Type I error is close to the nominal level with the Adjusted Q test. In addition, the power functions of the Adjusted Q test and the IPD method are nearly identical, and substantially higher than for the traditional Cochran’s Q. The Adjusted Q test therefore properly accounts for the dependence in test statistics across the SAT-rwCC comparisons, unlike the traditional Cochran’s Q.

### Impact of heterogeneity testing

#### Scenario 1: impact of heterogeneity testing in null scenario

In Table 2, we show the expected operating characteristics with a range of different RRs in the second rwCC. In the null scenario, where because of some bias, the RR in one of the two rwCC may be lower than the true value of 50%. As expected, when the response difference between the rwCCs increases, the Type 1 error, observed response difference, and probability of detecting heterogeneity also increase. However, given the low sample sizes in the rwCC, power is relatively low for detecting heterogeneity even at the relaxed p-value threshold of 0.10. A 30% RR difference is detected in only 64% of trials; the Type I error rate is 30% in the remaining 36% of trials where no heterogeneity is detected, leading to an overall Type I error rate of nearly 11% (30% * 36%). Thus, although heterogeneity testing successfully identifies some high-bias scenarios, in many reasonable scenarios it will be underpowered, leading to Type 1 error inflation (albeit less than if we always pool).

**Table 2:** Operating characteristics of a two-step procedure for deciding whether or not to pool

				Type I error		Response difference (%)	
SAT response (%)	rwCC #1 response (%)	rwCC #2 response (%)	Pooling probability^a^	Conditional on pooling	If always pool	Expected when always pooling	Average when pooling
50	50	50	0.94 (0.0008)	0.04 (0.001)	0.04 (0.0008)	0	0.01 (0.0003)
50	50	40	0.87 (0.001)	0.06 (0.001)	0.06 (0.001)	5	5.01 (0.0003)
50	50	30	0.67 (0.001)	0.14 (0.001)	0.13 (0.001)	10	10.35 (0.0004)
50	50	20	0.36 (0.002)	0.3 (0.002)	0.27 (0.002)	15	16.04 (0.0005)

^a^Data is pooled when heterogeneity is not detected

In these simulations, the RR in the SAT and in the first rwCC is 50%, and the RR in the second rwCC varies. The pooling probability is the probability that the heterogeneity test fails to reject (if it rejects, we do not pool). The Type I error rate is calculated under two different decision rules: we pool only when the heterogeneity test fails to reject (‘Conditional on pooling’), or we always pool (‘If always pool’). The average estimated response difference is also calculated (last column) and compared to the expected response difference when always pooling. Monte Carlo standard errors are in parentheses, for 100,000 replicates. 

Note that in each row of Table 2, the Type I error and response difference conditional on pooling are approximately equal to those of if we always pooled. This counterintuitive finding is because the average response across the two rwCC depends only weakly on whether the observed RRs are similar to each other or not, conditional on the true RRs in the two rwCC. For example, if the true RRs are 50% in rwCC #1 and 40% in rwCC #2, a typical case when similar RRs would be observed might be a rate of 45% in both datasets, while a typical case when different RRs would be observed might be a RR of 50% in one dataset and 40% in the other. In either case, the average rwCC RR is 45%, leading to Type I error inflation and bias in estimation of the response rate difference.

#### Scenario 2: impact of heterogeneity testing when treatment improves outcomes

In Scenario 2, we examine a situation where there should be a true RR difference between the SAT and both rwCCs, but the RR in one of the rwCCs is biased upward leading to Type 2 error. As shown in Table 3, the low power to detect heterogeneity means that the datasets will be inappropriately pooled often, leading to potentially severe underestimation of the treatment effect. For example, with a 20% RR difference between the rwCC, datasets will be pooled 67% of the time, and the average estimate of the treatment effect will be 20% when pooling, a third lower than the true RR difference of 30%.

**Table 3:** Operating characteristics of two-step procedure for deciding whether or not to pool

				Rejection probability (comparison to SAT)		Response difference (%)	
SAT response (%)	rwCC #1 response (%)	rwCC #2 response (%)	Pooling probability^a^	Conditional on pooling	If always pool	Expected when always pooling	Average when pooling
80	50	50	0.94 (0.0002)	0.85 (0.0001)	0.85 (0.0005)	30	30.01 (0.0001)
80	50	60	0.87 (0.0003)	0.70 (0.0002)	0.71 (0.0005)	25	24.94 (0.0001)
80	50	70	0.67 (0.0005)	0.50 (0.0003)	0.52 (0.0005)	20	19.68 (0.0001)
80	50	80	0.36 (0.0005)	0.27 (0.0005)	0.31 (0.0005)	15	13.96 (0.0001)

^a^Data is pooled when heterogeneity is not detected

In these simulations, the RR in the SAT is 80% and in the first rwCC is 50%, and the RR in the second rwCC varies. Monte Carlo standard errors are in parentheses, for 1,000,000 replicates.

## Discussion

This article makes several contributions to the existing literature on data pooling [6, 21, 23, 33]. First, we present a framework for data pooling focused on RWD, and specifically rwCC analyses. The three steps of this framework are: pre-specification, dataset selection, and outcomes analyses (including analysis of heterogeneity).

Second, we evaluate how best to detect heterogeneity in the rwCC use case and demonstrate that standard approaches to heterogeneity assessment are biased. To address this gap, we describe an Adjusted Cochran’s Q test, similar to existing methods in the literature for meta-analysis of dependent results, which accounts for correlation in the treatment estimates given the common SAT, and matches the statistical power of IPD heterogeneity testing. In scenarios in which all IPD can be combined, heterogeneity can be assessed either by directly comparing the rwCCs, or using the Adjusted Q. The former is the preferable approach; however, a use case in which only the Adjusted Q is feasible is when IPD may be available but not fully shareable across study sites. This can occur when IPD from the SAT can be combined with IPD from each rwCC, but IPD from different rwCCs cannot be combined (i.e., if one or more of the rwCCs is derived from a registry) [34]. If the sponsor wished to combine these results, this could be accomplished using a fixed effect meta-analysis approach, although adjustments (e.g., similar to those proposed for the Adjusted Q test) should be made to standard errors to account for non-independence of the results being meta-analyzed (see the Appendix for further discussion).

Third, our simulation results highlight the limitations of quantitative heterogeneity testing in protecting against Type 1 or Type 2 error especially in the presence of small sample sizes. While heterogeneity testing does identify scenarios with higher bias, its lack of statistical power when there are only a few rwCC (especially with the low sample sizes that would lead a sponsor to consider pooling) make it an insufficient mitigation on its own. This is in line with previous research illustrating the dangers of two-step procedures where fixed effects or random effects meta-analysis is selected based on a preliminary test of heterogeneity [35, 36]. We note that if the goal of pooling RWD were to achieve greater generalizability rather than a rwCC use case, heterogeneity would be expected (and potentially even desirable), and thus, testing would not be needed. The latter use case is out of scope for the current manuscript.

Together, these results underscore the importance of careful selection of datasets (‘what to pool’) based on clinical/data considerations, to minimize heterogeneity prior to any statistical testing. This remains an area in which further research is needed, given the multitude of considerations and difficulty testing the underlying assumptions [14]. Subsequent heterogeneity test results should be used descriptively to contextualize study findings. If statistical heterogeneity is detected or if qualitatively large but non-significant differences are seen, the investigators should present dataset-specific results, and conduct sensitivity analyses to investigate potential sources of heterogeneity [23].

## Conclusions

In conclusion, we have provided a methodological framework for pooling separate RWD sources, particularly applicable to the use case of designing a rwCC for a single arm trial in a rare subpopulation. Additionally, we provide simulations demonstrating the performance of a heterogeneity assessment methodology and describe considerations for this essential component of pooling. As researchers and regulators seek to benefit patients with novel insights from RWD pooled from multiple available sources, we hope the methods presented herein can enhance the rigor of such analyses.

### Supplementary Information

**Additional file 1: Appendix.**

## Data Availability

All data generated or analysed during this study are included in this published article and its supplementary information files.

## Abbreviations

RWD: Real-world data
rwCC: Real-world comparator cohort
SAT: Single-arm clinical trial
IPD: Individual participant data
RR: Response rate
