# Supplementary material for: Considerations for pooling real-world data as a comparator cohort to a single arm trial: a simulation study on assessment of heterogeneity
Source: BMC Med Res Methodol. 2023 Aug 24;23:193. doi: 10.1186/s12874-023-02002-7 (PMC10464044; doi:10.1186/s12874-023-02002-7)
Supplement: Supplementary file 1 — Additional file 1: Appendix. [file 12874_2023_2002_MOESM1_ESM.docx]

# Considerations for pooling real-world data as a comparator cohort to a single arm trial: a simulation study on assessment of heterogeneity

Daniel Backenroth^1^, Trevor Royce^2^, Jose Pinheiro^1^, Meghna Samant^1^, Olivier Humblet^2^

^1^Janssen Research & Development ^2^Flatiron Health, Inc.

**Corresponding author:**
Olivier Humblet
233 Spring Street
New York, NY 10013
ohumblet@gmail.com

##

## Appendix: Meta-analysis for dependent studies

We propose a methodology for carrying out a fixed effect meta-analysis of SAT-rwCC comparisons carried out on an aggregate level, that properly accounts for the dependence of these effect estimates caused by using the same SAT for all comparisons. A fixed effect model should be used since we expect the true effect size in each study being combined to be the same (since all effect modifiers should be adjusted for, ideally, and bias should be controlled).

In a fixed effect meta-analysis, when the independence assumption is satisfied, inverse-variance weights, which minimize the variance of the combined effect, are used (Borenstein et al.). For these inverse variance weights, the weight for the ith study is W_i_=1/V_i_, where V_i_ is the error variance for study i. The error variance of the combined effect when combining effects for k studies is then $V_{m}=1/\sum_{i=1}^{k} W_{i}$. When the independence assumption is not satisfied, different weights should be used. Using the method of Lagrange multipliers, we can show that if the covariance matrix of the effects for the studies is Σ, then the weights that minimize the variance of the combined effect are $\Sigma^{-1}1$. These are the column sums of the inverse of Σ. As in the independent case, we normalize the weights so that their sum is equal to 1, so that our weighted mean effect (if the effects are in the vector Y) is given by $M={1^{T}\Sigma^{-1}}Y/{1^{T}\Sigma^{-1}}1$. The variance of this weighted mean effect is $1/{1^{T}\Sigma^{-1}}1$, which is the inverse of the sum of all elements of the inverse covariance matrix $\Sigma^{-1}$.

Note that in some circumstances, the weights for a study could be negative, which cannot occur for standard inverse-variance weights. For example, assume we have two studies, the standard errors of the effect sizes in the two studies are $\sigma_{1}$and $\sigma_{2}$, and the correlation between the effect sizes is ρ>0, which we expect when we compare a single SAT to more than one rwCC. Then the covariance matrix of the study effects is

| ${\sigma_{1}}^{2}$ | ρ$\sigma_{1}\sigma_{2}$ |
| --- | --- |
| ρ$\sigma_{1}\sigma_{2}$ | ${\sigma_{2}}^{2}$ |

The inverse of this matrix is proportional to

| ${\sigma_{2}}^{2}$ | -ρ$\sigma_{1}\sigma_{2}$ |
| --- | --- |
| -ρ$\sigma_{1}\sigma_{2}$ | ${\sigma_{1}}^{2}$ |

Therefore, the weights will be proportional to ${\sigma_{2}}^{2}-$ρ$\sigma_{1}\sigma_{2}$ and ${\sigma_{1}}^{2}-$ρ$\sigma_{1}\sigma_{2}$. Therefore, if ρ is positive and greater than the smaller of $\sigma_{1}{/\sigma}_{2}$ and $\sigma_{2}{/\sigma}_{1}$, one of the weights will be negative. If the standard errors of the effects for the two studies are similar in size, then the correlation between the effects would need to be very high in order for one of the weights to be negative. If the standard errors of the effects for the two studies are very different in size, a small correlation between the effects would be enough in order for one of the weights to be negative.

## List of abbreviations

| RWD | real-world data |
| --- | --- |
| rwCC | real-world comparator cohort |
| SAT | single-arm clinical trial |
| IPD | individual participant data |
| RR | response rate |

## Code for heterogeneity simulation study

library(tidyverse)

library(data.table)

library(metafor)

library(knitr)

library(expm)

library(broom)

library(Barnard)

Q_res_file <- "q_test_evaluate.csv"

IPD_res_file <- "ipd_evaluate.csv"

PrettyResultsIPD <- function() {

rr <- fread(IPD_res_file)

p <- 0.05

summ <- rr[, .(power = mean(fisher <= p),

pooled_est_accept = 1 / n * mean((sa - (rw1 + rw2) / 2)[fisher > p]),

pooled_est_uncond = 1 / n * mean(sa - (rw1 + rw2) / 2)),

by = .(rw2_p, sa_p, rw1_p, n)]

summ[, `:=`(diff = rw1_p - rw2_p)]

p1 <- ggplot(summ %>% filter(diff >= 0),

aes(x = 100 * diff, y = 100 * power, color = as.factor(n))) +

geom_line() +

geom_point() +

ylab("Power (%)") +

xlab("Response rate difference (%)") +

theme_bw(13) +

scale_color_discrete("n")

ggsave("fisher_power.jpeg", height = 5, width = 6)

gathered <- summ %>%

select(diff, n, Conditional = pooled_est_accept,

Unconditional = pooled_est_uncond) %>%

gather(type, value, -diff, -n)

p2 <- ggplot(gathered %>%

mutate(n = factor(paste0("n=", n),

levels = paste0("n=", unique(.$n)))), aes(x = 100 * diff, y = 100 * value, linetype = type)) +

geom_line() +

facet_wrap(~n) +

scale_linetype_discrete("") +

theme_bw(13) +

xlab("Response rate difference (%)") +

ylab("Average bias from pooled analysis (%)")

ggsave("pooling_bias.jpeg", height = 5, width = 6)

}

PrettyResultsQ <- function(){

rr <- fread(Q_res_file)

summ <- rr[, .(Q = mean(qp_same <= 0.05),

IPD = mean(log_reg <= 0.05),

`Adjusted Q` = mean(qp_same_adjust <= 0.05)), by = .(rw2_p, rw1_p)] %>%

select(rw2_p, rw1_p, `Q`, `Adjusted Q`, IPD) %>%

gather(type, value, -rw2_p, -rw1_p) %>%

mutate(diff = 100 * (rw2_p - rw1_p),

value = 100 * value) %>%

filter(diff >= 0) %>%

mutate(type = factor(type, levels = c("Adjusted Q", "Q", "IPD")))

p <- ggplot(summ, aes(x = diff, y = value, col = type)) +

geom_line() +

geom_point() +

theme_bw(13) +

ylab("Probability of rejection (%)") +

xlab("Response rate difference (%)") +

scale_color_discrete("")

ggsave("q.jpeg", height = 5, width = 6)

print(p)

}

lr_func <- function(rw1, rw2, n) {

df <- data.frame(a = c(rw1, rw2), d = c(1, 0)) %>%

mutate(b = n - a)

mod <- glm(cbind(a, b) ~ d, data = df, family = binomial)

tidy(mod) %>%

filter(term == "d") %>%

pull(p.value)

}

f_func <- function(r1, r2, n1, n2) {

m <- matrix(c(r1, n1 - r1, r2, n2 - r2), nrow = 2)

fisher.test(m)$p.value

}

b_func <- function(r1, r2, n1, n2) {

m <- barnard.test(r1, n1 - r1, r2, n2 - r2)

m$p.value[2]

}

p_func <- function(r1, r2, n1, n2) {

prop.test(x = c(r1, r2),

n = c(n1, n2))$p.value

}

RunTrials <- function(sa_p, rw1_p, rw2_p, n_sa, n_rw1, n_rw2, reps) {

dat <- data.table(sa = rbinom(n = reps, size = n_sa, prob = sa_p),

rw1 = rbinom(n = reps, size = n_rw1, prob = rw1_p),

rw2 = rbinom(n = reps, size = n_rw2, prob = rw2_p))

dat[, `:=`(rw_sum = rw1 + rw2)]

dat[, `:=`(test_pool_vs_sat = p_func(r1 = sa, r2 = rw_sum,

n1 = n_sa, n2 = n_rw1 + n_rw2)),

by = .(rw_sum, sa)]

dat[, `:=`(test_rw_vs_rw = p_func(r1 = rw1, r2 = rw2, n1 = n_rw1, n2 = n_rw2)),

by = .(rw1, rw2)]

dat[, `:=`(pool_diff = sa / n_sa - (rw1 + rw2) / (n_rw1 + n_rw2))]

dat[, `:=`(sa_p = sa_p, rw1_p = rw1_p, rw2_p = rw2_p, n_sa = n_sa, n_rw1 = n_rw1,

n_rw2 = n_rw2, reps = reps)]

}

SummarizeScenarios <- function(res) {

summ1 <- res %>%

group_by(rw2_p) %>%

summarise(p_reject_uncondl = sum(test_pool_vs_sat < 0.05) / unique(reps))

summ <- res %>%

group_by(rw2_p) %>%

mutate(pool = test_rw_vs_rw > 0.1) %>%

filter(pool) %>%

summarise(p_pool = n() / unique(reps),

p_reject = sum(test_pool_vs_sat < 0.05) / n(),

mean_diff_pool = mean(pool_diff),

.groups = "drop") %>%

left_join(summ1, by = "rw2_p") %>%

transmute(`rwCC #2 response (%)` = rw2_p * 100,

`Pooling prob.` = p_pool,

`Type I error (when pooling)` = p_reject,

`Type I error (if pool all the time)` = p_reject_uncondl,

`Response difference (when pooling) (%)` = 100 * mean_diff_pool)

return(summ)

}

# power.prop.test(p1 = 0.8, p2 = 0.5, sig.level = 0.05, power = 0.9)

Scen_Null <- function(n = 50) {

set.seed(1)

reps <- 100000

res <- map_dfr(c(0.5, 0.6, 0.7, 0.8), #seq(0.5, 0., by = 0.1),

.f = function(rw2_p) {

RunTrials(sa_p = 0.5, rw1_p = 0.5, rw2_p = rw2_p, n_sa = n, n_rw1 = n / 2, n_rw2 = n / 2, reps = reps)

})

summ <- SummarizeScenarios(res)

kable(summ, format = "rst", digits = 2) %>%

print()

}

Scen_Alt <- function() {

set.seed(1)

reps <- 1000000

res <- map_dfr(seq(0.5, 0.8, by = 0.1), .f = function(rw2_p) {

RunTrials(sa_p = 0.8, rw1_p = 0.5, rw2_p = rw2_p, n_sa = 50, n_rw1 = 25, n_rw2 = 25, reps = reps)

})

summ <- SummarizeScenarios(res) %>%

rename(`Rejection prob. (when pooling)` = "Type I error (when pooling)",

`Rejection prob. (if pool all the time)` = "Type I error (if pool all the time)")

kable(summ, format = "rst", digits = 2) %>%

print()

}

EvaluateIPD <- function() {

S <- seq(0.2, 0.8, by = 0.1)

grid <- expand_grid(sa_p = 0.5,

rw2_p = S,

n = c(25, 50, 75, 100)) %>%

mutate(rw1_p = sa_p)

res <- pmap_dfr(grid, function(sa_p, rw1_p, rw2_p, n){

dat <- RunTrials(sa_p = sa_p, rw1_p = rw1_p, rw2_p = rw2_p, n_sa = n, n_rw1 = n,

n_rw2 = n, reps = 5000)

})

write.csv(res, file = IPD_res_file, row.names = F)

}

RiskDiff <- function(m_1, n_1, m_2, n_2){

p1 <- m_1 / n_1

p2 <- m_2 / n_2

y <- p1 - p2

v <- p1 * (1 - p1) / n_1 + p2 * (1 - p2) / n_2

return(list(y = y, v = v))

}

GetCovar <- function(rw, sa, n) {

sa_boot <- rbinom(n = 1000, size = n, prob = sa[1] / n)

rw1_boot <- rbinom(n = 1000, size = n, prob = rw[1] / n)

rw2_boot <- rbinom(n = 1000, size = n, prob = rw[2] / n)

rw1 <- RiskDiff(rw1_boot, n, sa_boot, n)

rw2 <- RiskDiff(rw2_boot, n, sa_boot, n)

cov(cbind(rw1[[1]], rw2[[1]]))

}

EvaluateQ <- function(s = seq(0.5, 0.8, by = 0.1)) {

res <- map_dfr(s, function(rw2_p){

sa_p <- 0.5

rw1_p <- 0.5

n <- 100

reps <- 1000

dat <- data.table(sa1 = rbinom(n = reps, size = n, prob = sa_p),

sa2 = rbinom(n = reps, size = n, prob = sa_p),

rw1 = rbinom(n = reps, size = n, prob = rw1_p),

rw2 = rbinom(n = reps, size = n, prob = rw2_p))

q_func <- function(rw, sa, covar_adjust = F) {

if (covar_adjust) {

r1 <- RiskDiff(rw[1], n, sa[1], n)

r2 <- RiskDiff(rw[2], n, sa[2], n)

covar <- GetCovar(rw, sa, n)

cov_inv_sqrt <- solve(sqrtm(covar))

new_stats <- cov_inv_sqrt %*% c(r1$y, r2$y)

rma <- rma.uni(yi = new_stats, vi = c(1, 1))

} else {

rma <- rma.uni(ai = rw, bi = n - rw, ci = sa, di = n - sa,

measure = "RD")

}

return(rma$QEp)

}

dat[, `:=`(qp_same = q_func(c(rw1, rw2), c(sa1, sa1))), by = .(rw1, rw2, sa1)]

dat[, `:=`(qp_same_adjust = q_func(c(rw1, rw2), c(sa1, sa1), covar_adjust = T)), by = .(rw1, rw2, sa1)]

dat[, `:=`(qp_diff = q_func(c(rw1, rw2), c(sa1, sa2))), by = .(rw1, rw2, sa1, sa2)]

dat[, `:=`(log_reg = lr_func(rw1, rw2, n)), by = .(rw1, rw2)]

dat[, `:=`(rw2_p = rw2_p, sa_p = sa_p, rw1_p = rw1_p, n = n, reps = reps)]

})

write.csv(res, file = Q_res_file, row.names = F)

return(res)

}

CorrelationAverageAndDifference <- function(){

mmm <- map_dfr(1:10 %>% set_names(.), function(x){

p1 <- 0.3

p2 <- 0.5

n <- 100

v1 <- p1 * (1 - p1) / n

v2 <- p2 * (1 - p2) / n

corr <- ( v1 - v2) / (v1 + v2)

print(corr)

ns <- 500000

r1 <- rbinom(ns, size = n, prob = p1)

r2 <- rbinom(ns, size = n, prob = p2)

dd <- data.table(r1 = r1, r2 = r2)

dd[, `:=`(ave = (r1 + r2) / 2,

diff = r1 - r2)]

print(cor(dd$ave / n, dd$diff / n))

f_func <- function(r1, r2) {

m <- matrix(c(r1, n - r1, r2, n - r2), nrow = 2)

fisher.test(m)$p.value

}

dd[, `:=`(fisher = f_func(r1, r2)), by = list(r1, r2)]

dd[, `:=`(fisher_sig = fisher <= 0.05)]

dd[, .(m = mean(ave)), by = fisher_sig]

}, .id = "i")

m[fisher_sig == F, mean(m)]

}
